# Single-cell analyses of circulating tumor cells

**DOI:** 10.7497/j.issn.2095-3941.2015.0056

**Published:** 2015-09

**Authors:** Xi-Xi Chen, Fan Bai

**Affiliations:** Biodynamic Optical Imaging Center, School of Life Science, Peking University, Beijing 100871, China

**Keywords:** Circulating tumor cells (CTCs), single cell sequencing, metastasis

## Abstract

Circulating tumor cells (CTCs) are a population of tumor cells mediating metastasis, which results in most of the cancer related deaths. The number of CTCs in the peripheral blood of patients is rare, and many platforms have been launched for detection and enrichment of CTCs. Enumeration of CTCs has already been used as a prognosis marker predicting the survival rate of cancer patients. Yet CTCs should be more potential. Studies on CTCs at single cell level may help revealing the underlying mechanism of tumorigenesis and metastasis. Though far from developed, this area of study holds much promise in providing new clinical application and deep understanding towards metastasis and cancer development.

## Introduction

Most cancer-related deaths are caused by metastasis. The presence of circulating tumor cells (CTCs) in peripheral blood was first observed in 1869^1^; this phenomenon is an important “intermediate step” in cancer metastasis^1^. Studies on CTCs not only reveal the critical biological processes involved in cancer metastasis but also provide a non-invasive method for cancer detection, diagnosis, prognosis, and test of drug response. Research on CTCs has increased in recent years because of the vital role of CTCs in cancer metastasis and significant potential in clinical applications. In this review, we present a summary of these studies, with specific emphasis on single-cell analyses of CTCs.

## CTCs and metastasis

**Figure 1:**
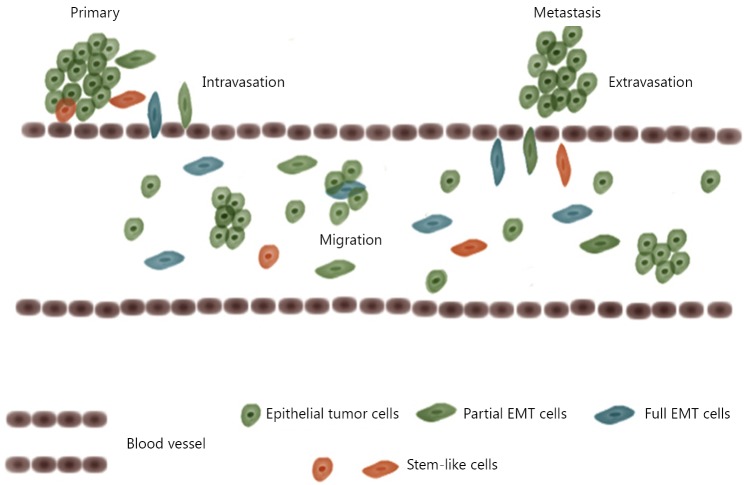
CTCs and metastasis. Tumor cells may transform to mesenchymal-like cells and intrude the blood vessel wall, entering blood streams. Tumor cells migrate as single or in microemboli. When located in appropriate distal sites, cells escape from blood streams (mesenchymal to epithelial transition maybe involved) and seed new tumor lesions. The population of CTCs includes both epithelial and mesenchymal-like cells, non-stem and stem-like cells.

A century has passed since the first observation of CTCs in cancer patients’ blood. The characterization of cancer metastasis has greatly advanced over time. The key steps can be summarized as intravasation, migration, and extravasation (**Figure 1**).

When metastasis initiates, the tumor cells in the primary site proliferate, thereby attracting blood vessels to provide nutrients and oxygen for tumor growth while giving the tumor cells a chance to invade the blood vessels^2^. Epithelial to mesenchymal transition (EMT) is speculated to play a critical role in this intravasation process. To intrude blood vessels, epithelium-originating tumor cells should lose cell polarity and tight junctions among different cells, thus forming spindle-like, mesenchymal-shaped cells and acquiring a higher motility power to break the defense line of the basement membrane and vascular wall^3^^-^^5^. Many cell surface markers, including the most commonly applied E-cadherin, vimentin, and fibronectin, are used to characterize the EMT process. Whether EMT is required in metastasis remains unclear. Several researchers reported that tumor cells cooperate with mesenchymal cells rather than transit into them to complete the metastasis process^6^. CTC is an ideal location to provide understanding on the issue, and direct investigations on CTCs mostly support the existence of this transition process^7^^-^^11^. Researchers identified epithelial and mesenchymal markers on CTCs, thereby suggesting that some CTCs express both markers, and they are in a partial EMT state^12^.

Studies on CTCs can also provide clues on tumor initiation. CTCs represent a population capable of immigration and tumor initiation. A stem-like subpopulation exists in the tumor tissue, which possesses high ability of tumorigenesis^13^^-^^15^ (**Figure 1**). For CTCs to seed metastasis, the entire process should have extremely low efficiency^16^. Even if CTCs succeed in intravasation, most of them cannot survive the tough environment in the blood stream and eventually die from anoikis. One of the main goals of CTC research is to identify the stem-like subpopulation within CTCs, which has real tumorigenesis ability. A successful characterization of these cells will provide significant clinical values.

CTCs sometimes aggregate to form microemboli (circulating tumor microemboli, CTM). In the early 1970s, researchers proved that injections of tumor cells in the form of microemboli into mouse significantly increase the efficiency of tumor initiation^17^^,^^18^. CTM may endow tumor cells advantages in survival and help to avoid anoikis. Meanwhile, the participation of mesenchymal cells may provide a “moving niche”, which gives CTCs strong viability and moving ability^12^. Tumor cells have the most effective initiation ability when they cooperate with mesenchymal cells^19^. Previous studies showed that when a combination of two tumor cell lines with clearly different metastatic abilities is injected into a mouse, approximately 90% of the metastatic tumors are multiclonal, whereas a cell line with low-metastatic ability can only form less than 10% of the metastasis when injected alone^12^^,^^20^. This result suggests that CTM formation could even increase the metastasis of weak metastatic tumor cells.

For several decades, strategies for metastasis research are mainly based on mouse models or cell lines. The lack of proper objects for research extensively limited further investigation. To date, studies on CTCs have created a new opportunity; CTCs are the true link between primary and metastatic tumors. These cells may carry valuable information of both primary and metastatic sites, as well as specific details of intravasation, migration, and extravasation.

## Methods for CTC detection and enrichment

**Figure 2:**
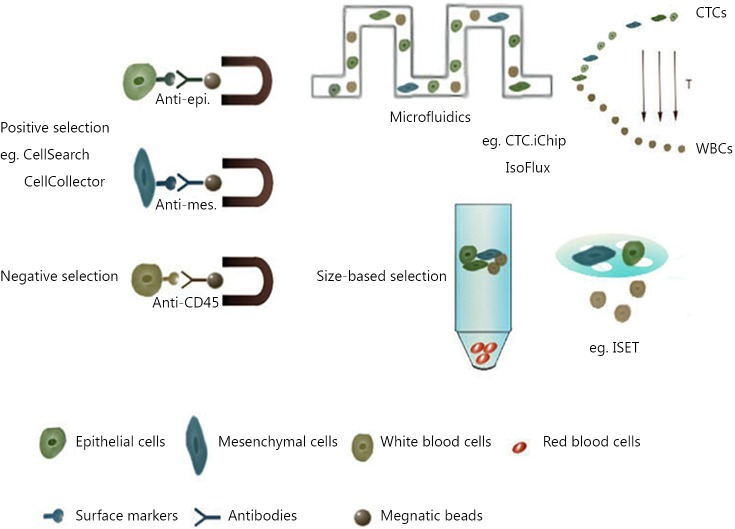
Methods for CTCs enrichment. CTCs enrichment can be based on their biological properties (e.g., surface markers) or physical properties (e.g., size). Surface marker-based methods include positive selection and negative selection. For CTCs detection, most applied marker in positive selection is EpCAM, and in negative selection is CD45. Subsequent enrichment can be managed by centrifugation or size-based filtration. Microfluidic-based enrichment can help to improve the sensitivity and recovery of target cells and can combine selection methods using both biological and physical properties.

The main challenge in CTC research is their detection and enrichment, which requires the ability to detect one CTC out of almost 10^9^ normal blood cells^21^. Based on the known properties of tumor cells, numerous platforms for CTC detection and enrichment were developed and can be classified into two major categories: (I) immunochemistry-based methods, including positive selection (enrichment of epithelial marker-positive cells) and negative selection (depletion of CD45-positive blood cells); and (II) physical property-based methods (selection by cell size or electrical charge) (**Figure 2**).

Epithelial-marker based approaches are the most widely applied strategies for CTC detection. Among all the platforms, CellSearch from Janssen Diagnostics is considered the most successful. CellSearch is the only FDA-approved platform for CTC detection in clinical practice on patients with breast, prostate, and colorectal cancers. The practicability of CellSearch has been widely verified by many studies and referred as the “golden standard” for evaluating newly developed approaches^22^. CellSearch uses magnetic beads coupled with anti-EpCAM antibodies at the initial step to enrich EpCAM-positive epithelial cells and later stain the enriched cells for Cytokeratin (CK), CD45, and nucleus (DAPI) to capture CTCs (CK+CD45-DAPI+) and remove leukocytes (CK-CD45+DAPI+).

Currently, no other epithelial marker-based approach has performed better than CellSearch, but several platforms that implement microfludics have great potential. IsoFlux is a commercially availabe platform for CTC detection. This platform also relies on EpCAM-coupled-magnetic beads to capture CTCs, but the capture step is conducted in a microfluidic chip that can increase the chance to arrest CTCs with lower EpCAM expression. Both EpCAM^low^ (MDA-MB-231) and EpCAM^high^ (SKBR3)-expressing tumor cells can be recovered with a successful rate of 74% and 85%, respectively^23^. Moreover, application of a combination of different epithelial markers (for example, EpCAM and MUC1) may also help to recover more epithelium-originating tumor cells^24^. The limitation of blood volume can also be a contributing factor to the low yield of CTC capture. CellCollector was developed to resolve this problem. This platform uses an EpCAM antibody-coated wire to capture CTCs *in vivo*^25^. However, metastasis often involves EMT; thus, epithelial marker-dependent approaches may miss numerous CTCs that have low or absent epithelial marker expression. Considering this phenomenon, the depletion of blood cells may be a supplementary method.

A chip-based platform CTC-iChip shows a good example of a combination of size-based selection and label-dependent enrichment^26^. This platform has positive (^pos^CTC-iChip) and negative selection modes (^neg^CTC-iChip). CTC-iChip initially applies a micro-column device to remove erythrocytes and cell fragments. Subsequently, the remaining mononuclear cells go through a second inertial focusing microfluidic device for further separation of EpCAM+ and CD45+ cells. Authors emphasized that even for several low-EpCAM-expressing cell lines, ^neg^CTC-iChip still achieved a high recovery. ISET^27^ is another widely accepted size-based approach. This platform applies a specific membrane filter for tumor cell selection because tumor cells are often larger and stiffer than blood cells. Other size-based platforms include the ScreenCell and CanPatrol^28^. The main advantage for using membrane filter is that CTM can be retained for further investigation. Nonetheless, the sizes of tumor cells may vary significantly and not be always larger than blood cells because of the broad heterogeneity among tumor cells. Therefore, these size-based platforms need careful clinical validations. Furthermore, cells sticking on the filter are sometimes inconvenient for further manipulation; new devices were invented to address this problem, such as the Parsotix^29^ and JETTA^30^ systems.

## Current clinical applications of CTCs

**Figure 3:**
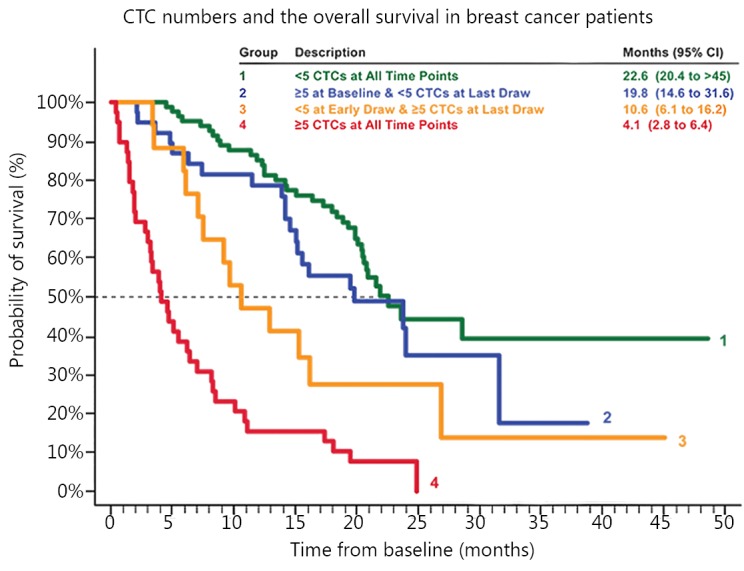
CTCs and prognostic prediction. The number of CTCs detected per 7.5 mL blood shows a strong correlation with the survival rate in breast cancer. If more than 5 CTCs detected, the survival rate is lowered to <50%. Similar results have been obtained in prostate cancer, colorectal cancer and lung cancer. (Modified from Janssen Diagnostics, LLC, 2015).

To date, CTC enumeration has been widely used as a prognostic marker for patients’ overall survival rate. CellSearch is the only FDA-approved system for CTC detection applicable in clinical practice. A cut-off value of ≥5 (breast and prostate cancers) or ≥3 (colorectal cancer) in 7.5 mL blood is highly significant in predicting worse prognosis (**Figure 3**) (modified from Janssen Diagnostics, LLC, 2015)^31^^-^^33^. Counting the number of CTCs also shows predictive power in lung cancer^34^, melanoma^35^, head, and neck carcinoma^36^, as well as pancreatic cancer^37^. Even for several early-stage cancers, such as colorectal and breast cancers, CTC numeration also displays potential value in prognostic prediction^38^^,^^39^.

In addition to its role in prognosis prediction, changes in CTC number during medical treatment can also deliver valuable information of therapy response. Patients with a sharp decrease in CTC count after treatment often show better outcomes^33^^,^^40^. Accordingly, CTC enumeration can also help guide drug development^41^^,^^42^.

## Single-cell analyses of CTCs

Besides enumeration, CTC characterization at the molecular level is also in progress. Key genetic variation events that are well-demonstrated in solid tumors are also assessed in CTC analysis. For example, in FISH and quantitative PCR implementation, researchers evaluate the TMPRSS2-ERG fusion, AR gain, and PTEN loss in CTCs, primary tumor, and metastatic site of prostate cancer patients^43^; the fusion event of TMPRSS2-ERG in CTCs is also used as a biomarker for sensitivity of abiraterone acetate treatment in castration-resistant prostate cancer patients^44^. Furthermore, the HER2 overexpression, which is an important determinant of therapy, in breast cancer can also be evaluated in CTCs at certain time points^45^. Another study conducted on lung cancer patients focused on the mutational status of the drug resistance-related gene EGFR in CTCs. Researchers collected CTCs from 12 patients, and 11 of them had activated mutations on EGFR in CTCs. Some of the mutations in CTCs seemed to be *de novo*, indicating that the mutations could not be detected in the primary tumor^46^. These studies demonstrate the potential use of CTCs as a real-time monitoring of response to therapy and guide in therapeutic planning.

The rapid development of next-generation sequencing is accompanied by progress in genomic and transcriptomic characterization of CTCs to obtain a comprehensive understanding of a specific population of tumor cells. Meanwhile, sequencing-based analyses can identify novel genetic variation events during tumor development. Yu *et al*.^8^ conducted the first transcriptome sequencing of isolated CTCs and identified some of the EMT-related genes with abnormal expression, including TGF-β and FOXC1, thereby suggesting the dynamic change of epithelial and mesenchymal composition. These results are in accordance with the observed RNA-FISH and immunofluorescence results.

CTCs are generally rare in patients’ blood; one of the main challenges for sequencing-based analyses is to exclude the contamination from white blood cells (WBCs). CTCs can be separated from WBCs according to surface markers by using flow cytometry or micromanipulation. However, this phenomenon brings another critical problem, that is, the remaining CTCs are insufficient to provide DNA or RNA for the next generation sequencing. Given the progress in developing whole genome amplification and whole transcriptome library construction, sequencing-based analyses for CTCs can be scaled down to single-cell level. In 2009, Tang *et al*.^47^ improved the protocol for mRNA library construction to enable the mRNA-Seq of one single cell. Since the study was conducted, single-cell transcriptome analysis has been widely used.

The first single-cell transcriptome analysis of CTCs was conducted with the Smart-Seq method for full-length mRNA-Seq^48^. Researchers compared the libraries generated from six CTCs from a melanoma patient with those of other melanocyte-originating cells (including primary melanocytes and melanoma cell lines), as well as blood, immune, and embryonic stem cells. The results proved the melanoma origin of the identified NG2+ CTCs. Moreover, the results identified important plasma membrane proteins with significant gain or loss of expression, including CDH1 and HLA1.

Whole genome amplification has always been a challenging step for any genomic analysis at the single-cell level. To date, the dominant method for single-cell genome amplification is multiple displacement amplification (MDA)^49^^,^^50^, which uses phi-29 polymerase, producing long amplicons in a strand-displacing method at 30 °C. MDA provides relatively high fidelity in amplified products and is widely applied to the mutation detection of single cells. Nevertheless, the amplification bias of MDA is severe, which may cause distortion of copy number determination and allele dropout. In 2012, Sunny Xie’s group^51^ developed multiple annealing and looping-based amplification cycles (MALBAC). This method significantly reduces the amplification bias and provides a high coverage and evenness across the whole genome.

**Figure 4:**
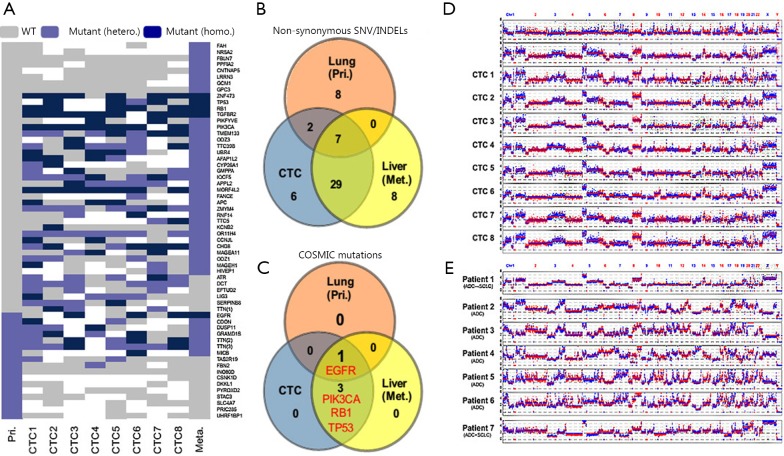
Genome features of single CTCs from lung cancer patients. CTCs were isolated from different lung cancer patients and the whole genome was amplified for sequencing. (A) Exome sequencing results suggested heterogeneity in SNVs of single CTCs from an individual patient. (B,C) Critical tumor mutations related to drug target, drug resistance, phenotypic transition can be detected in CTCs, including mutations on EGFR, PIK3CA, RB1, and TP53. (D) Differed from SNVs, the global CNV pattern is highly reproducible among CTCs from the same individual. (E) Even from different patients, CTCs share a relatively similar global CNV pattern. (Modified from Ni *et al*.^52^).

The first whole-genome analysis for CTCs was published in 2012. In this work, Ni *et al*.^52^ applied MALBAC for the whole-genome amplification of single CTCs. In this pioneering work, CTCs from seven lung adenocarcinoma patients (including a patient with a combination of adenocarcinoma and small cell lung cancer, and a patient with phenotypic transition from adenocarcinoma to small cell lung cancer) and four patients with small cell lung cancer carcinoma were isolated by CellSearch System. Subsequently, whole exome sequencing was performed on each single CTC to detect single nucleotide variations (SNVs), and low-depth whole-genome sequencing (~0.1×) was used to detect the copy number variations (CNVs). Ni *et al*.^52^ reported that SNVs detected in single CTCs are highly heterogeneous. They also reported that the important mutations associated with drug target (EGFR), drug resistance (PIK3CA), and phenotypic transition (TP53, RB1) can all be detected in CTCs, thus implying the significant clinical value of CTC genomic analyses. Moreover, Ni *et al*.^52^ found that regardless of the extensive heterogeneity in SNVs, almost all the CTCs from the same patient represented a reproducible CNV pattern. In lung adenocarcinoma patients, similarity in CNV pattern among different patients is also higher than expected. However, patients with SCLC exhibited distinct CNV pattern in their CTCs (**Figure 4**). Ni *et al*.^52^ also reported that reproducibility in the CNV patterns of CTCs remains stable at different time points during treatment in one SCLC patient.

Similar works are also conducted on colorectal cancer^53^. Researchers isolated 37 intact CTCs from six patients with stage IV colorectal carcinoma by using CellSearch System; and a limited MDA was applied to amplify the whole genome. The amplified DNA was then subjected to comparative genome hybridization microarray for copy number evaluation, as well as massive parallel sequencing of a panel of 68 colorectal-associated genes for mutation detection. Many important genetic variation events previously reported in colorectal carcinoma are also detected in CTCs, including mutations in APC, KRAS, and PIK3CA. Previous studies also verified by targeted deep sequencing that most of the seemingly exclusive mutations in CTCs are also present in primary tumor at a relatively low frequency in minor subclones. Steinert *et al*.^54^ also found that immune escape pathways are up-regulated in CTCs of colorectal cancer patients, thereby implying a mechanism for CTC survival in the bloodstream.

Lohr *et al*.^55^ recently proposed a validated pipeline for whole exome sequencing of single CTCs. Isolated CTCs were amplified for the genome, and libraries were then constructed. Using low-depth sequencing, some poorly qualified libraries were eliminated; finally, by combining different whole exome libraries in one patient, they obtained a relatively accurate spectrum of mutations. However, this combination of libraries ignored the heterogeneity among different CTCs in an individual patient, which may also play important roles in metastasis.

Compared with blood samples collected directly from cancer patients, mouse models may be a better alternative for the biological characterization of CTCs. In certain cancer types, CTCs are difficult to isolate, which makes mouse model advantageous in these types of cancer. Ting *et al*.^56^ applied their developed CTC-iChip device on an engineered pancreatic cancer mouse model (KPC) to isolate 168 single CTCs from five mice and managed to perform RNA-seq on 45% of the single CTCs. They found that CTCs show high level of multiple gene transcripts in the ECM pathway, which is normally expressed in reactive stromal cells rather than in epithelial cancer cells, including Dcn and Sparc. Sparc is a well-known ECM protein found in the stroma of primary human pancreatic ductal adenocarcinoma (PDAC). Ting *et al*.^56^ also verified that SPARC overexpression may enhance the metastasis in pancreatic cancer by using shRNA-mediated knockdown test in two human PDAC cell lines with relatively high SPARC expression (PDAC2 and PDAC3).

Many factors may influence the single CTC analysis results. For example, in choosing platforms for CTC enrichment, EpCAM-based platform usually provide the highest capture purity, which would lead to minor blood cells contamination. But they are only suitable with those high-epithelial-marker-expressing CTCs. Negative selection or size-based selection may provide a broader range of CTCs detection, thus would be more proper when heterogeneity among different CTCs is emphasized. However, negative selection is often accompanied by higher rate of normal cells contamination, thus it requires further careful identification of “real” CTCs. Microfluidic devices may also help to increase the sensitivity of low-epithelial-marker-expressing CTCs detection. For transcriptome analysis, it requires cells to be intact, thus platforms causing much damage to the cells are not suitable. Besides enrichment methods, whole genome amplification methods or library construction methods should also be carefully selected according to different purpose of research. Taking the whole genome amplification as an example, MALBAC performs better for CNV capture, while MDA is more accurate for SNV detection.

Research on CTCs at single cell level holds much promise because of the heterogeneity among CTCs. Polzer *et al*.^57^ reported that there are pre-existing CTCs resistant to ERBB2-targeted therapies, whose exploration may guide personalized treatment decisions. Yet currently the application of single CTC analysis in clinic is far from developed, mainly due to the limitation of CTC enrichment and isolation. Rare platforms could adapt to all types of cancer and the situations among different patients are highly diverse, making it hard to generally evaluate the efficiency or accuracy of single CTC analysis in clinic for now.

## Conclusion and perspectives

Although CTC research is still in its infancy, it is a promising field of study because of its scientific significance in elucidating cancer metastasis and clinical value in non-invasive cancer detection, prognosis, and diagnosis.

Combining the advanced sequencing methods, CTC analyses show an apparent advantage over tumor tissues. The access to CTCs is much easier and less invasive than that to tumor tissues, which can only be obtained from surgery or biopsy samples. However, the current detection and isolation of CTCs are the bottleneck for CTC analyses. More works are needed to validate the efficiency of the newly developed platforms. Over the past decade, significant improvements have been made. Thus, we are highly confident that the sensitivity and efficiency of CTC detection will be improved in the near future to provide a solid base for the downstream genomic and functional analyses.
